# Two Cases of the Destructive Effects of a Foreign Body Admitted into the Trachea, &c.

**Published:** 1810-10

**Authors:** Robert Kinglake

**Affiliations:** Taunton


*Two Cases of the destructive Effects of a foreign Body admitted into the Trachea, &c.* Communicated by Dr. Kinglake.

CALAMITOUS instances are not unfrequently occurring, of substances accidentally escaping into the trachea, and baffling the expulsive efforts of both nature and art, in endeavouring to effect their removal. Within a short time, two distressing cases of this kind have come within my knowledge, occasioned, in both instances, by a small dry bean falling into the trachea. The immediate inconvenience produced by these accidents, was that of insufferable irritation in the trachea, accompanied with anxious and laborious respiration. This state would often proceed to paroxysms of vehement coughing; but these struggles, after exhausting the afflicted patients, hurrying the action of the heart and arteries to a state of tremor, and bathing the surface in cold sweat, would subside, until the recruited powers of life led to a renewal of the convulsive but unavailing effort to detach and expel the offending body. In this unalleviated and hopeless career of affliction both patients survived a few days the recurrence of their respective accidents, when they fell hapless victims to the slow-paced approaches of suffocation tion. The effects of vomiting and coughing, exerted under every advantage of position, where wholly unsuccessful.

In dissection, the destructive beans were found in both cases at the lower part of the trachea, firmly fixed by the enlarged dimensions which they had acquired from vegetative expansion. Thus circumstanced, it was physically impossible to disengage them by any action which the trachea could exert on the obstructing bulk. In similar occurrences, therefore, it becomes both a chirurgical and physiological question of much practical importance to determine, whether it would not be adviseable to endeavour to remove such causes of inevitable destruction, by dividing the trachea sufficiently to admit of a search for such a substance by a suitable instrument, or even by the point of the fore finger. Such an opening would at once afford the operator free access to the extraneous body, and a way by which it may be discharged by natural effort. If the obstructing substance be dislodged from the situation to which inflammatory excitement, (through the medium of effused coagulable lymph), as well as its own enlarged dimensions, may have fettered it, its final removal will be much facilitated. Examples of dividing the trachea in rash attempts at suicide, have been known to occur, in which respiration has, for a time, been carried on through the aperture made in the trachea instead of by the mouth; and afterwards, by gradually and continually closing up the incised opening, the breathing has resumed its natural course. If this can happen under the rudest circumstances of self-violence, it surely may be more commodiously and securely effected by the address and management of surgical skill. *Anceps remedium melius quam nullum*, should be the justifying maxim on this occasion. Without an attempt to aid in the way proposed, the unhappy subjects of the accident under consideration must necessarily, in a large proportion, if not uniformly, fall victims to the suffocating difficulties of the case.

The practice of opening the trachea in enginal and other obstructions threatening suffocation, is not new. Fabescius ab Aqua pendente, speaks of it in the following terms:—"Plures auctores sectionem asperæ arteriæ probarunt, tam antiqui tam recentiones, etenim Abbucasis expressè dixit, quod in sectione asperæ arteriæ non est timor, idque comprobat exemplo ancillæ, quæ, cum aliquando sibi ipsi cultello asperam arteriam præcidisset ipsam sanavit facilè, et sine ullo periculo; sed et recentiones multi, ut Brasavolus, et alii ad ipsum attestantur. Cui opinioni ego libenter benter subscribo." Then describing the mode of making the proposed incision, he says,—" Inclinato ægroti capite retrorsum, ut arteria reddatur conspicua, addo ego, et arteria distendatur, et longior fiat et membranosa intermedia, seu interstitia magis appareant, transversa linea incidamus cutem quæ exterius in collo est, inter duos circulos, ita ut membranam secemus, mediam inter cartilaginem ipsam, non cartilaginem; quæ sectio facienda est infra caput asperæ arteriæ, spatio trium ejusdem quatuorve circulerum."—Although the admonition here cited is not expressly given for the extraction of foreign bodies from the tracheal tube, yet its admissibility on such occasions is obviously implied. Indeed those instances would seem more especially to justify incurring whatever hazard may attend the operation, in as far as no disease exists independently of that which is induced by the direct irritation of mechanical pressure, and the exclusion of a due influx of atmospheric air into the lungs. This view of the subject then presents a solemn appeal to the medical world, whether the proposal here suggested should not be early resorted to, in preference to abandoning the afflicted patient to the destructive torments of incessant, painful, and fruitless coughing.

I am, &c.

ROBERT KINGLAKE.

*Taunton, Sept.* 6, 1810.

P. S. Please to notice an *erratum* in your Number 138, for August, viz. page 109—line 8—for *secretions*, read *accretions*.

